# Machine Learning Approaches for Geospatial Modeling of Urban Land Surface Temperature: Assessing Geographical Compactness, Interpretability, and Causal Inference

**DOI:** 10.3390/s25175380

**Published:** 2025-09-01

**Authors:** Nhat-Duc Hoang

**Affiliations:** 1 Institute of Research and Development, Duy Tan University, Da Nang 550000, Vietnam; hoangnhatduc@duytan.edu.vn; 2 Faculty of Civil Engineering, Duy Tan University, Da Nang 550000, Vietnam

**Keywords:** land surface temperature, urban morphology, geographical compactness, machine learning, SHAP, Landsat 8, Sentinel-2, highland region, Quang Ngai Province, Vietnam

## Abstract

This study presents a data-driven framework for modeling urban heat in a highland region of Quang Ngai Province, Vietnam—an area with limited prior research on heat stress. Using advanced machine learning methods, including Category Boosting (CatBoost) and deep convolutional neural network (CNN), the spatial distribution of urban land surface temperature (LST) is predicted based on topographical, land use/land cover, urban morphological, proximity, and compactness features. Our findings show that incorporating urban compactness metrics significantly enhances prediction accuracy, with CatBoost explaining 89% of LST variance. Based on Shapley Additive Explanations, built-up density, bare land density, distance to river, green space density, and built-up cluster compactness are identified as the most influential factors. Machine learning-based causal analysis further clarifies the direct effects of key urban features on LST. The proposed framework helps reveal distinct characteristics of the study area with respect to urban heat properties. The research findings can support sustainable urban planning and heat stress alleviation in the study area.

## 1. Introduction

Urban expansion is converting natural landscapes into built-up environments worldwide. According to the United Nations [1], in 2018, 55% of the world’s population lived in urban areas; this proportion is projected to reach 68% by 2050. The global urban population grew from 751 million in 1950 to 4.2 billion by 2018, with Asia accounting for 54% of this total. Europe and Africa each make up 13% of the global urban population. Urban area cover is projected to increase by 1.2 million km^2^ by 2030; this change will almost triple the total urban area compared to the year 2000 [2].

The expansion of built-up areas causes local temperatures to rise and intensify the urban heat island (UHI) effect [3]. In Vietnam, rapid urbanization, changes in land use and land cover (LULC), and climate change have contributed to higher land surface temperatures (LST) and increased heat stress in many cities. The frequency and severity of heat stress and drought are increasingly reported in the region [4,5]. In [6], daily maximum temperature data from 102 meteorological stations between 1980 and 2020 were analyzed. The authors also found that the lagged effects of the El Niño–Southern Oscillation (ENSO) largely drive these heatwave patterns in Vietnam.

According to [7], Vietnam has experienced a sharp rise in both the frequency and severity of heatwaves in recent decades. Analyses in [8] found a positive correlation between LST and housing density. In [4], the research indicated that climate change, especially during the hot and humid summer months, has led to an increase in the number of hot days. Consequently, both the frequency and intensity of heat stress have increased; this fact leads to more severe public health impacts.

Moreover, it is evident that developing countries face many challenges in managing urban heat stress [9]. These challenges stem from rapid population growth, constrained budgets, and inadequate urban planning tools. As a result, regional authorities need cost-effective solutions for planning and monitoring. Advanced tools are essential for developing sustainable strategies, managing land use, and expanding green spaces. In Vietnam and other developing countries, limited financial resources hinder large-scale fieldwork for assessing urban LST. Publicly available LST data from Landsat 8 imagery and the Google Earth Engine platform offer a practical solution to these challenges [10]. Open-source LST data enables quick assessment of the UHI effect and identification of urban heat hotspots [3,10,11,12].

The Central Highlands of Vietnam is a mountainous region (500–1500 m high) with a diverse minority population. Despite recent economic growth, this area still struggles with limited infrastructure and urban planning challenges. Rapid changes in land use and population growth put additional pressure on urban planning and environmental management. This situation highlights the need for in-depth spatial modeling and analysis of LST to support sustainable urban planning and urban heat stress mitigation.

In recent years, Geographic Information System (GIS) and geospatial analyses based on retrieved LST data have become important tools for understanding how urban form affects local temperatures [13]. These methods enable researchers to map and analyze the spatial distribution of LST. This capability is crucial for revealing patterns linked to LULC and urban morphological features. Using GIS, LST data can be overlaid with information on built-up areas, green spaces, and other features to identify zones most impacted by the urban heat island (UHI) effect [14]. Geospatial analysis can also be used to compare the effects of different urban forms on temperature; such a comparison provides evidence for better planning decisions [15]. Advanced GIS techniques, including spatial modeling and machine learning, can enhance modeling accuracy. Overall, GIS and geospatial analysis are essential for urban planning and for tackling increased urban heat stress [16].

Spatial modeling of LST in urban areas is challenging due to its complex interactions with LULC, environmental factors, and urban morphology. LST patterns depend on multiple intermingled factors, such as green cover, built-up density, impervious surfaces, and local topography [14,17,18]. Disentangling these interactions requires advanced geospatial analysis and models. Despite progress in using topographic factors and urban morphology for LST modeling [19,20,21], non-linear relationships and spatial variability remain major obstacles. These challenges highlight the need for advanced data-driven tools for large-scale urban heat stress analysis.

Machine learning tools are essential for handling the complex, non-linear relationships between temperature patterns and influencing factors. Advanced algorithms can analyze large, multivariate datasets and utilize up-to-date LST and remote sensing data to reveal hidden patterns [19,22,23,24]. Previous studies show that methods like gradient boosting [19,25], ensemble models [26,27], and deep learning [18] predict LST with high accuracy and can quickly assess urban heat stress at large scales.

Among these, artificial neural networks (ANNs) [28] and deep neural networks (DNNs) [18] have consistently demonstrated good predictive accuracy, especially when diverse datasets are available. These deep learning models excel in capturing non-linear interactions between LST and a multitude of influencing variables. Support Vector Machines (SVM) has been successfully employed for LST modeling due to their robust regression capabilities stemming from the principle of margin maximization [28,29]. Random forest [28] and gradient boosting variants [18,19,26] have also demonstrated robust performance; these models can deliver high prediction accuracy along with efficient and rapid model training.

However, the major limitation of ANNs and DNNs lies in their “black box” nature, which can hinder the explicit contribution of each input factor to the prediction. The training phase of an SVM involves solving a quadratic programming problem, which can become computationally intensive as dataset size grows. This makes SVMs less practical for large-scale GIS datasets compared to tree-based ensemble methods or deep learning approaches. The performance of SVM-based models is also highly sensitive to the selection and tuning of kernel functions used during the model’s training and prediction phases. Additionally, random forest and gradient boosting methods models cannot handle categorical variables natively. These models can also be susceptible to overfitting if they are not tuned appropriately.

In the field of machine learning, Category Boosting (CatBoost) and deep convolutional neural networks (CNNs) are particularly powerful. CatBoost handles both real-valued and categorical data with strong resilience against overfitting [30], while CNNs excel at detecting complex patterns in data [31]. These methods are well-suited for modeling the diverse factors shaping urban LST. Particularly, CatBoost is well-suited for LST modeling in urban environments due to several distinct advantages over existing methods. CatBoost can natively handle categorical features without requiring data preprocessing or encoding. This capability is useful in geospatial datasets, where categorical data—such as LULC—can be critical predictors. Additionally, CatBoost incorporates advanced techniques to reduce prediction bias and overfitting.

To accurately capture the spatial variation of urban LST, it is crucial to generalize the relationship between this variable and the current status of urban growth. Urban growth typically expresses three main spatial patterns: infilling, edge expansion, and outlying development [32]. Infilling tends to create a more regular and cohesive urban landscape, while edge expansion results in a fragmented and complex pattern. Outlying growth, on the other hand, increases the density and diversity of urban patches. Generally, these processes of urban expansion can modify the compactness property of a city, as they often lead to a more dispersed urban form [33].

The overall urban form is crucial for understanding urban heat stress. As pointed out in [34], more compact urban forms can intensify the urban heat environment. In urban studies, the compactness index, such as the Polsby–Popper Compactness Index [35], is a useful tool for studying urban characteristics. It measures how closely the shape of an urban area resembles a circle, which is considered the most compact form. This compactness index helps compare the urban form in different neighborhoods and facilitate the understanding of urban landscape and urban sprawl phenomenon [36].

Causal inference is valuable in urban LST studies, as it enables researchers to quantify the direct effects of various factors on urban heat. Unlike simple correlation analysis, causal inference estimates how changes in governing factors like urban greenness, impervious surfaces, or urban morphology actually cause changes in LST [37,38]. Recent research has integrated machine learning with causal inference to assess the impact of urban features and interventions on the urban environment [39,40]. This combined approach allows for precise modeling of how specific changes in controlled variables affect urban thermal properties.

Based on the literature review, several research gaps in the study of urban LST and heat stress, especially in the context of developing regions like the Central Highlands of Vietnam, can be stated as follows:

(i) First, while many studies examine how environmental variables and urban features affect LST [13,19,41,42], few have focused on the role of geographical compactness, such as how local compactness features of urban components influence LST patterns.

(ii) Second, although various machine learning methods have been used to deal with the task of interest [24], there is still no clear agreement in the research community on the most effective approach for predicting urban LST. In particular, powerful methods like CatBoost and CNN, which have shown great success in other complex modeling tasks, have not yet been widely applied to LST prediction.

(iii) Third, the application of causal inference methods to urban LST studies is still limited and requires further exploration. Machine learning-based causal inference is a recently emerged field. This field of study is important for identifying the direct effects of different variables and supporting better decision-making for urban planning [37,38,39].

(iv) Fourth, there is a lack of detailed records and research on urban heat stress in the Central Highlands of Vietnam.

(v) Finally, it remains unclear which environmental and urban morphology factors are most important for LST modeling in the aforementioned region. Therefore, the use of big data and advanced tools, such as Shapley Additive Explanations (SHAP), could significantly help address this gap.

**Figure 1:**
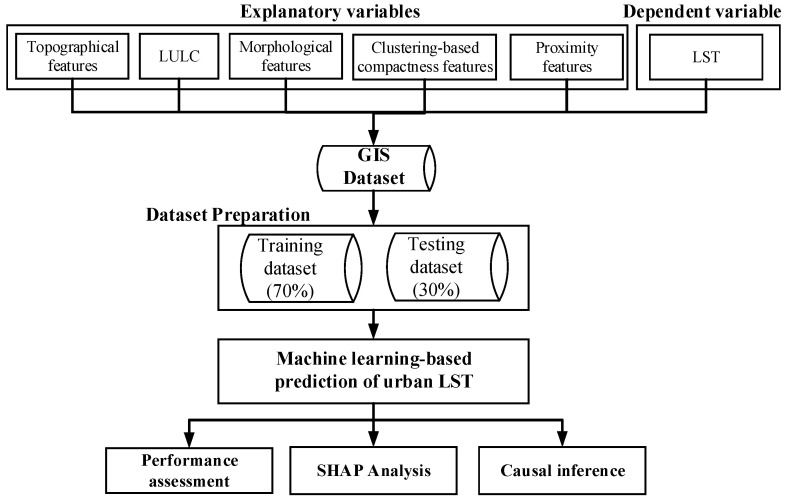
Workflow for machine learning-based urban LST modeling and interpretation.

Hence, the primary goal of this study is to collect LST data and other remotely sensed variables for the urban area in the highland region of Quang Ngai Province, Vietnam. The overall workflow of this study is summarized in Figure 1. We aim to assess the current status of urban heat stress in the region during cloudless days of the dry season in 2024 by conducting analyses on LST from satellite imagery. Our research puts forward a novel machine learning-based modeling framework that utilizes CatBoost and CNN to predict and analyze urban LST variation. We incorporate a set of diverse explanatory variables, including topographical, urban morphological, and proximity features, as well as introduce clustering-based metrics to represent urban compactness characteristics. The current study evaluates the usefulness of these urban compactness features through the Wilcoxon signed-rank test. Furthermore, the Shapley Additive Explanations (SHAP) method is used to improve model interpretability. Machine learning-based causal inference is also employed to quantify the direct effects of urban features on LST variation. Via these advancements, our study aims to provide an integrated framework that supports urban heat mitigation and sustainable development in the study area.

Notably, the distinguishing methodological advance of this study is the integration and comparison of CatBoost and deep CNN within a unified framework for urban LST prediction. This framework is designed for application in a study area with unique geospatial and urban morphological characteristics. CatBoost can provide superior prediction performance due to its efficient encoding of features, robust generalization, and ability to mitigate overfitting. Meanwhile, the deep CNN is adapted to exploit complex spatial patterns present in the input data. Additionally, this work introduces an innovative approach that incorporates clustering-based compactness metrics as new features to represent urban form.

## 2. Research Method and Materials

### 2.1. Study Area and the Remote Sensing Data

**Figure 2:**
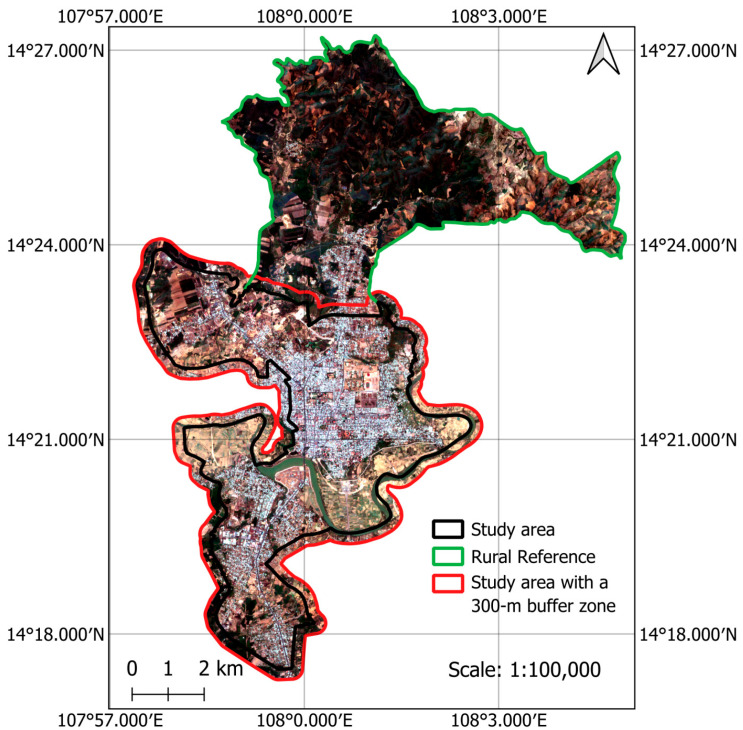
The study area (true color composite of Sentinel-2’s bands).

An urban center (refer to Figure 2) in the southern region of Quang Ngai, Vietnam, is selected as the study area of the current work. This area lies in the Central Highlands region of Vietnam at an elevation ranging from 469 to 588 m above sea level. It contains the wards of Dak Bla, Kon Tum, and the south section of the Dak Cam ward. This urban area is currently experiencing a period of rapid urbanization. Due to its location in a low-lying valley, the region experiences low humidity, and its average annual temperature is relatively higher than neighboring regions. In recent years, there has been an increase in the frequency and severity of heatwaves in the region due to the effect of climate change [7,43]. It is noted that to evaluate the UHI effect, the northern section of the Dak Cam Ward is used as a rural reference. Moreover, to eliminate the effect of pixels at the edge on the spatial analysis results, a 300 m buffer zone is created around the study area boundary, as demonstrated in Figure 2. The data within this buffer zone are excluded from both the training and testing phases of the machine learning models.

This study utilizes multiple remote sensing datasets, as outlined in Table 1. LST in the study area was obtained from Landsat 8 imagery and accessed through the Google Earth Engine (GEE) code editor. Notably, as pointed out in [44], urban heat stress is a major concern during the periods of droughts and heat waves. The UHI effect can be intensified in the dry season. Therefore, UHI coupled with intense heat waves has direct impacts on human health. In the Central Highlands of Vietnam, the dry season typically occurs from January to March and in December. Accordingly, this study examines urban LST during these dry months; herein, we focus on the dry season of 2024 as a case study.

LST values used as the dependent variable in this study were obtained from the atmospherically corrected surface temperature band (ST_B10) within the Landsat 8 Collection 2 Level-2 dataset. Cloud masking based on the QA band and median filtering was applied to the imagery. The Normalized Difference Vegetation Index (NDVI) was used in the process of LST calculation; this index was generated using the SR_5 and SR_6 bands. Elevation data is retrieved from NASA’s SRTM dataset [45] and processed in GEE to generate the topographical maps for the study area. For LULC classification in 2024, Sentinel-2 imagery was analyzed using a random forest classifier within the GEE platform. All bands of Sentinel-2 were standardized to a 10 m resolution in GEE. The bands originally at 10 m (i.e., B2, B3, B4, and B8) retain their native resolution, while the bands with a native resolution of 20 m (i.e., B5, B6, B7, B8A, B11, and B12) were upsampled to 10 m using the nearest neighbor resampling method. The maps presented in this research were standardized to a spatial resolution of 30 × 30 m and processed using the open-source QGIS software (version 3.34.10) (https://qgis.org/).

### 2.2. Land Surface Temperature Retrieval and Assessment of Heat Stress

**Figure 3:**
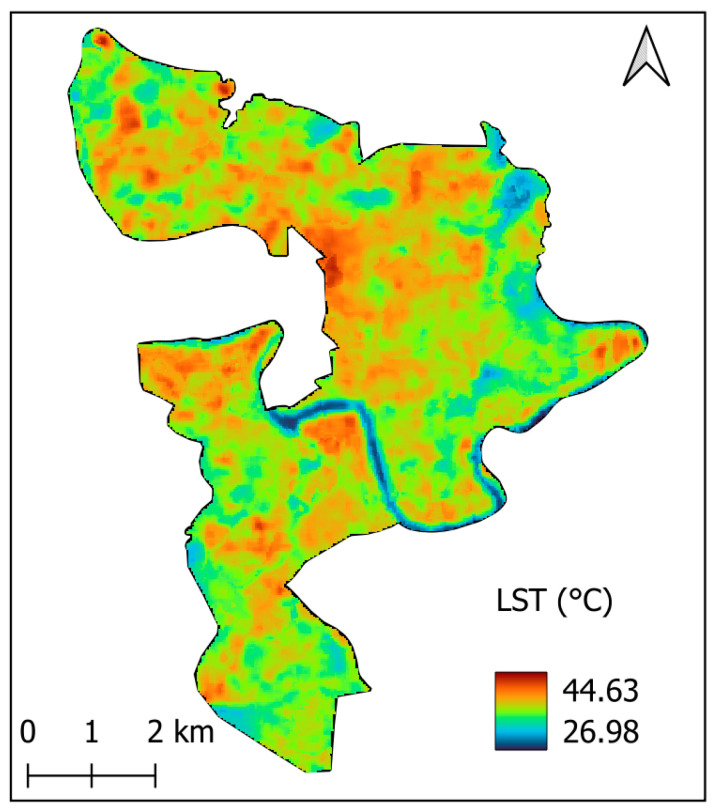
Spatial distribution of median LST values in the study area, derived from cloud-free Landsat 8 images acquired during the dry season of 2024.

As mentioned earlier, the LST dataset is derived from the surface temperature band (ST_B10) of the Landsat 8 Level-2 product. It is noted that the original thermal data acquired by the TIRS sensor on Landsat 8 has a native spatial resolution of 100 m. In the Level-2 product, these thermal measurements are resampled to 30 m to facilitate integration with higher-resolution multispectral data; however, the effective spatial accuracy remains limited to the original 100 m resolution. The 30 m output reflects interpolated values and should not be interpreted as a true enhancement of spatial detail in thermal signals. Figure 3 presents the median LST for each pixel within the study area during the dry season of 2024. The use of the median helps capture the typical LST over the observation period and minimizes the influence of outliers in the data.

To calculate LST, the process involves converting the values from the Landsat 8 spectral band into spectral radiance, as described in the following steps [11,46]:

(1) $$T_{B}=MRF+B_{10}\times ARF$$

where *B*_10_ denotes the digital number of the 10th band; MRF (0.0003342) and ARF (149) represent the multiplicative and additive rescaling factors, respectively.

Furthermore, the emissivity-corrected LST is computed in the following equation [47]:

(2) $$T_{S}=\frac{T_{B}}{1+(\lambda \times T_{B}/\rho )\times \ln(\varepsilon )}-273.15$$

where *T_S_* is the estimated surface temperature measured in Celsius (°C); λ (10.8 µm) denotes the wavelength of emitted radiance; ρ=h×c/b (1.438 × 10^−2^ mK), where h is Planck’s constant (6.626 × 10^−34^ Js), c represents the velocity of light (2.997 × 10^8^ m/s), and *b* is Bolzmann’s constant (1.38 × 10^−23^ J/K); the factor of 273.15 is used for converting the temperature from Kelvin (K) to Celsius (°C); and ε is the land surface emissivity computed as follows [48]:

(3) $$\varepsilon =0.004\times P_{\upsilon }+0.986$$

where $P_{\upsilon }$ is the vegetation proportion.

The vegetation proportion ($P_{\upsilon }$) is calculated as follows [11]:

(4) $$P_{\upsilon }={\left(\frac{NDVI-NDVI_{\min}}{NDVI_{\max}-NDVI_{\min}}\right)}^{2}$$

where NDVI, NDVI_min_, and NDVI_max_ denote the value of NDVI, minimum NDVI, and maximum NDVI values, respectively.

The magnitude of the UHI effect can be calculated as the difference in temperature between the urban center and a rural reference as follows [49,50]:

(5) $$UHI_{ij}=T_{U}-{\bar{T}}_{R}$$

where $UHI_{ij}$ represents the magnitude of the UHI effect at coordinates (*i*,*j*), and $T_{U}$ and ${\bar{T}}_{R}$ are the temperature at coordinates (*i*,*j*) in the urban center and the mean temperature of the rural reference. Herein, the northern section of the Dak Cam Ward is employed as the rural reference.

Additionally, the UHI Effect Intensity (UHIEI) can be calculated to quantify the magnitude of the urban heat stress [51,52]. The UHIEI is calculated as follows:

(6) $$UHIEI=\frac{T_{U}-{\bar{T}}_{R}}{{\bar{T}}_{R}}$$

**Figure 4:**
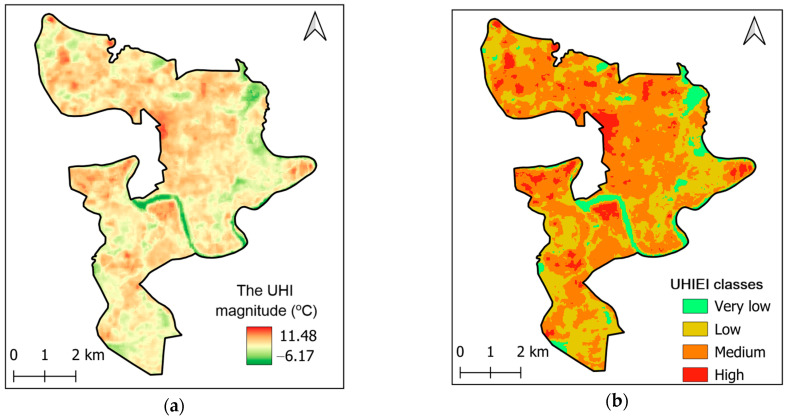
UHI effect in the study area, derived from the median of cloud-free Landsat 8 images acquired during the dry season of 2024: (**a**) UHI effect magnitude; (**b**) the UHIEI classes.

Figure 4a illustrates the spatial distribution of UHI magnitude across the study area, with values ranging from approximately −6.17 °C to 11.48 °C. As can be seen in the figure, most of the urban area experiences positive UHI values, indicating that it is generally warmer than the rural reference. Moreover, the highest UHI magnitudes are concentrated in the central and northern parts of the study area; this fact indicates intense heat stress in these zones. The UHIEI is demonstrated in Figure 4b. It is noted that Figure 4a and Figure 4b, respectively, represent the median UHI magnitude and UHIEI in the study area during the time period of interest.

**Figure 5:**
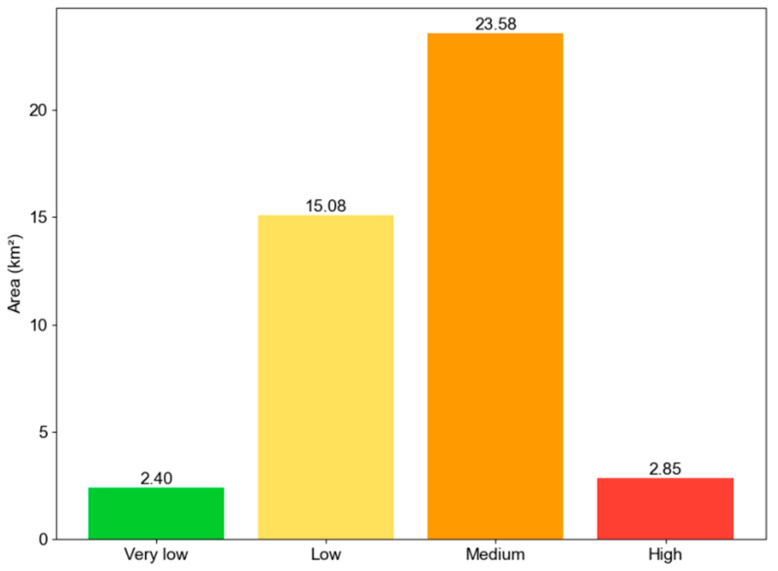
Area of UHIEI classes in the region.

The UHIEI index is classified into 5 levels as recommended in [51]: no UHI effect (UHIEI ≤ 0.0), low (0.0 < UHIEI ≤ 0.1), medium (0.1 < UHIEI ≤ 0.2), high (0.2 < UHIEI ≤ 0.3), and extremely high (UHIEI > 0.3). As observed from the figure, the majority of the study area falls within the medium and high UHIEI classes; this fact indicates widespread and significant urban heat stress. The area of each UHIEI zone is summarized in Figure 5. The class of medium UHIEI covers the largest area, with 23.58 km^2^; this category accounts for the dominant heat intensity zone in the urban area. Low UHIEI is the second-largest class, which occupies 15.08 km^2^. The area of high UHIEI is 2.85 km^2^; this area signifies critical hotspots with the most intense urban heat stress. The class of very low UHIEI only occupies 2.40 km^2^. In general, the data points out that the medium UHIEI class dominates the landscape, and moderate heat stress is widespread across the region. High UHIEI zones, though limited in area, should be located since they are critical for urban planning and heat mitigation due to their potential impact on public health and thermal comfort.

### 2.3. Remote Sensing-Based Feature Selection

Explanatory variables play a crucial role in the spatial modeling of urban LST. It is because these variables help to identify, quantify, and interpret the factors that influence the local climatic patterns across a study area. To analyze the spatial variation of the urban LST in the urban center, a set of explanatory variables is selected to construct the GIS dataset. The variable selection process relies on both a review of previous research and the data availability in the region. The employed remote sensing variables in this study are summarized in Table 2.

Urban LST is strongly influenced by a combination of environmental, LULC, morphological, and proximity features. Built-up density and bare land density tend to increase LST, as impervious and exposed surfaces absorb and retain more heat, while high green space density tends to reduce LST through shading and evapotranspiration. Topographic factors such as elevation, slope, and aspect can further influence temperature patterns [53,54,55]. In addition, proximity to cooling features like green spaces and rivers plays a significant role. In general, areas farther from these features typically have higher LST due to a lack of natural cooling. Urban morphological features, particularly the compactness of built-up clusters, can intensify heat retention by restricting airflow and concentrating heat-absorbing surfaces.

#### 2.3.1. Topographical Features

**Figure 6:**
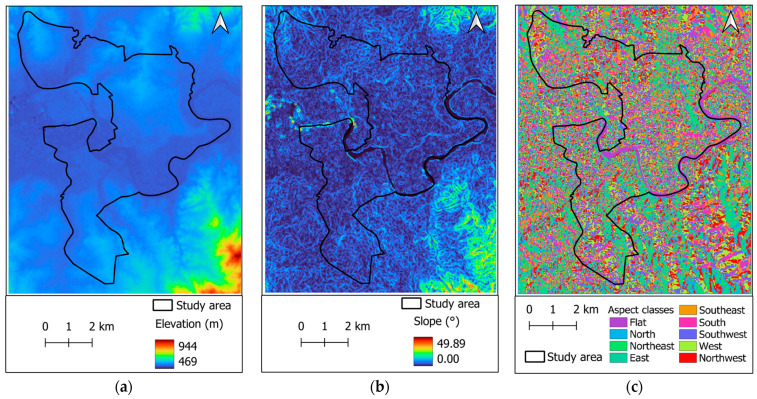
Topographic features: (**a**) elevation, (**b**) slope, and (**c**) aspect.

Topographical features (as shown in Figure 6), including elevation, slope, and aspect, play a key role in determining solar radiation exposure and local climatic conditions; therefore, they impose a direct effect on urban surface temperatures [56,57]. Elevation is important for capturing LST patterns in the urban center. Generally, areas at higher elevations exhibit lower surface temperatures. Moreover, elevation can also interact with other environmental factors, such as proximity to water bodies and vegetation cover. Slope affects solar radiation exposure of an area. Steeper slopes often receive less direct sunlight, and flatter areas may absorb more solar energy. Additionally, aspect provides information about the orientation of a slope; hence, this factor is also relevant in LST modeling [19].

#### 2.3.2. LULC and Density-Related Features

**Figure 7:**
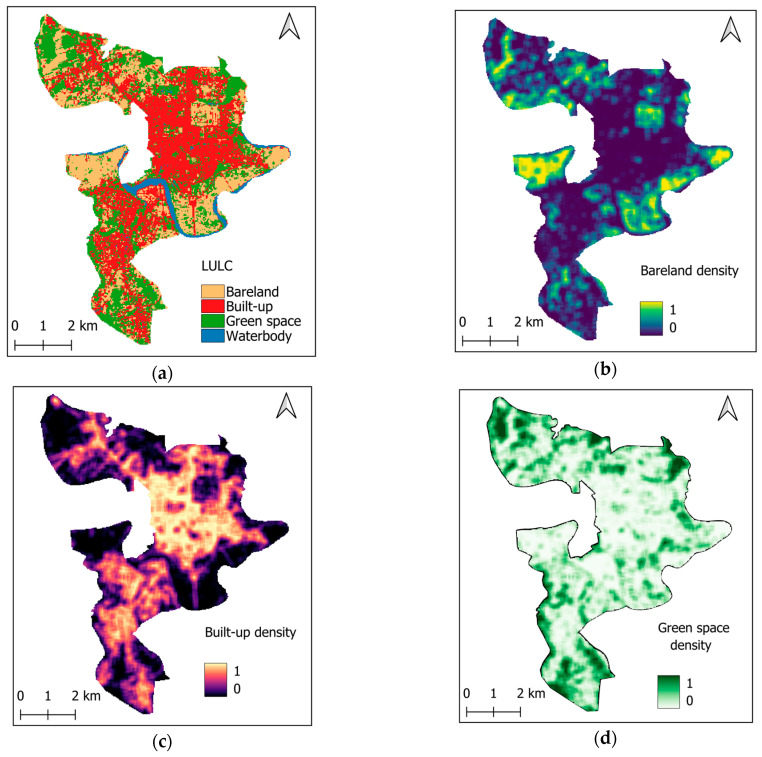
LULC and morphological features: (**a**) LULC map in 2024, (**b**) bare land density, (**c**) built-up density, and (**d**) green space density.

LULC mapping is also an important task in predicting urban LST [58,59,60]. It is because different surface types (e.g., built-up areas, vegetation, water bodies, and bare land) exhibit distinct thermal behaviors. Built-up and impervious surfaces tend to absorb and retain more heat. Meanwhile, areas with vegetation and water bodies generally have lower temperatures due to evaporation and moisture content. This study uses random forest (RF) classifier and Sentinel-2’s spectral bands to construct the LULC map of the study area in 2024. The data labeling and classification processes are conducted in the GEE platform. A dataset consisting of 2000 samples is collected within the study area. The data in each class (i.e., bare land, built-up, green space, and water body) contains 500 sampling points. The ground truth label of the data points is verified via Google Earth Pro. The training-to-testing ratio of this dataset is 85/15. The RF model is constructed with 500 individual decision trees. After the training process, this classifier achieves satisfactory performance, with an overall classification accuracy of 95%. The classification accuracy rates for the bare land, built-up, green space, and waterbody are 98.44%, 88.89%, 95.29%, and 97.01%, respectively. The resulting LULC map for the study area in 2024 is illustrated in Figure 7a. In this map, the areas of bare land, built-up, green space, and waterbody are 11.50, 17.89, 13.03, and 1.48 km^2^, respectively.

Additionally, urban morphological features play an important role in determining the thermal environment in cities [22,60,61]. The high density of built-up and bare land is typically associated with high urban LST [60]. Meanwhile, the denser the urban green spaces are, the more apparent their cooling effect is [62]. Hence, this study relies on the variables of bare land density, built-up density, and greenspace density to reflect the urban landscape composition in the study area. The density features are computed via a morphological filter with the size of 3 × 3 pixels (or 90 × 90 m). These variables help characterize local features that influence how heat is absorbed and retained across different sections of the urban landscape. The urban morphological features are demonstrated in Figure 7b–d.

#### 2.3.3. Proximity Features

**Figure 8:**
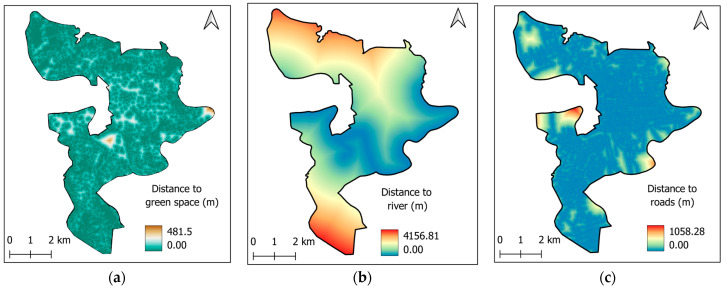
Proximity maps: (**a**) distance to green space, (**b**) distance to river, and (**c**) distance to roads.

Proximity variables, including the distance to green spaces, rivers, and roads, also play a significant role in governing the environmental impact of urban landscapes. Specifically, green spaces and rivers have been shown to provide considerable cooling effects and thermal comfort to mitigate heat stress [63,64,65]. Meanwhile, roads in the study area are usually made from asphalt concrete; this material typically absorbs more heat than the natural surfaces. As a result, roads are able to retain a large amount of solar radiation and experience a significant increase in temperature [66]. Road data is extracted from OpenStreetMap via https://extract.bbbike.org (accessed on 3 June 2025). The maps showing proximity variables are demonstrated in Figure 8.

#### 2.3.4. Geographical Compactness Assessment Based on k-Means Clustering and the Polsby–Popper Index

It is noted that LST is influenced by the spatial configuration of the urban landscape. Hence, urban compactness can be useful for landscape characterization and LST modeling. The compactness index generally measures how closely the shape of an urban patch resembles an ideal compact form (i.e., a circle). Compact urban forms can exhibit different thermal behaviors compared to sprawling or fragmented ones. In this study, we focus on the built-up and green space pixels in the LULC map to investigate the characteristics of urban compactness in the study area.

**Figure 9:**
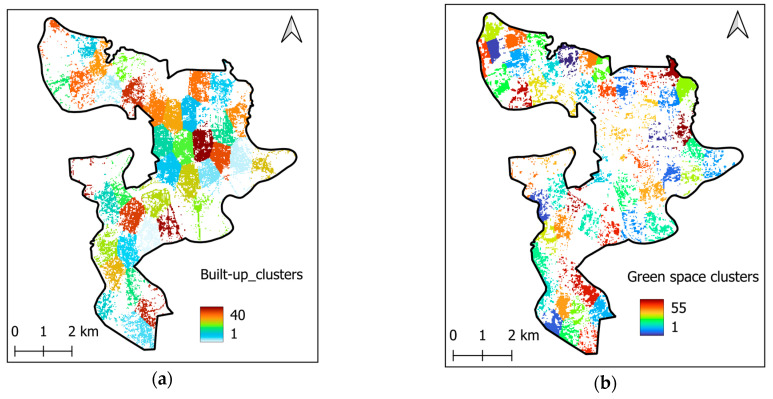
*k*-means clustering result: (**a**) built-up clusters and (**b**) green space clusters.

**Figure 10:**
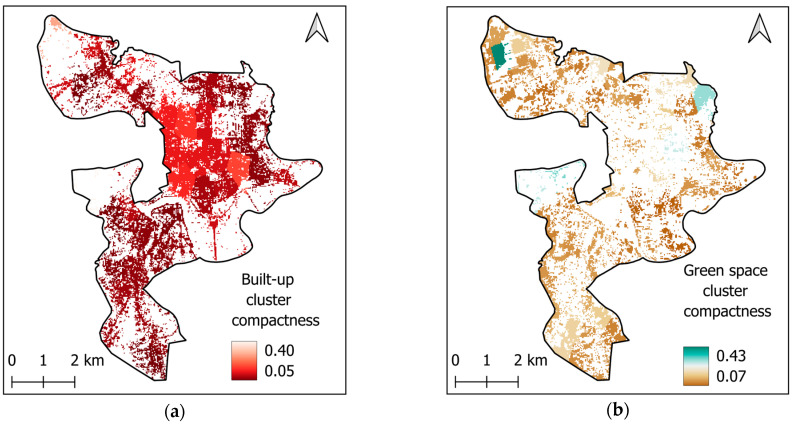
Geographical compactness analysis: (**a**) built-up areas and (**b**) green space areas.

The overall workflow of the urban compactness assessment can be described as follows:

(i) First, filtering operators are used to extract built-up and green space pixels from the original LULC raster file. This process yields two binary maps that represent the spatial distribution of these two LULC classes.

(ii) Second, spatial clustering analysis is performed on the newly constructed built-up and green space maps using the *k*-means algorithm. The *k*-means algorithm has been applied to group spatially distributed data in landscape ecology and urban studies [67,68]. This method groups the built-up green and space pixels into clusters that reflect coherent urban patches. Herein, the silhouette score [69] is employed to evaluate clustering performance and select the suitable number of clusters. The clustering outcomes are demonstrated in Figure 9.

(iii) Finally, the geographic compactness of each identified cluster is quantified using the Polsby–Popper index [35]. The geographic compactness maps are provided in Figure 10.

The Polsby–Popper index is a metric for measuring geographic compactness and has been employed in urban studies [36,70]. This index compares the area and perimeter of each cluster to assess its degree of compactness. The Polsby–Popper score ranges from 0 to 1; values closer to 1 indicate greater compactness. The Polsby–Popper index is calculated as follows:

(7) $$CI_{C}=\frac{4\pi A_{C}}{P_{C}^{2}}$$

where *CIc*, *Ac*, and *Pc* are the compactness index, area, and perimeter of a cluster *c*, respectively.

### 2.4. Modeling Approach

#### 2.4.1. CatBoost Regressor

Categorical Gradient Boosting (CatBoost) [30] is a popular supervised machine learning approach capable of solving complex function approximation problems by employing symmetric decision trees. CatBoost relies on the gradient boosting framework, which constructs an ensemble of predictors through the process of gradient descent in a functional space. This method can help construct robust predictive models via an iterative process of combining weaker base predictors.

At the level of individual learners, the CatBoost algorithm implements gradient boosting using binary decision trees. These trees are constructed by recursively partitioning the feature space into separate regions (or tree nodes) based on the values of selected splitting attributes. Each terminal node (or leaf) of the tree is assigned a value that is essentially an estimate for the response variable.

Notably, CatBoost can help address the occurrence of prediction shift in conventional gradient boosting decision tree algorithms. This phenomenon can lead to unstable gradient estimation and prediction performance. CatBoost deals with the issue of prediction shift by employing an innovative ordered boosting framework. This approach reduces bias in gradient estimation, as well as the complexity of the algorithm. Hence, this approach demonstrates strong performance when working with large and complex datasets [71,72,73].

Let $D={\left\{(x_{k},y_{k})\right\}}_{k=1,2,\ldots ,n}$ denote a dataset, where $x_{k}$ is a feature vector of *m* elements and $y_{k}$ is the target output (i.e., the urban LST at a certain location). A gradient boosting procedure aims to iteratively build a sequence of approximators *F^t^* to attain an accurate prediction of $y_{k}$ based on $x_{k}$ as follows:

(8) $$F^{t}=F^{t-1}+\alpha h^{t}$$

where α denotes the step size and *h^t^* is a based predictor (e.g., a decision tree) chosen from a family of models *H*.

The training phase of the CatBoost aims to minimize the following expected loss:

(9) $$h^{t}=\arg\min L(F^{t-1}+h)=\arg\min E(L(y,F^{t-1}(x)+h(x)))$$

To solve the above optimization problem, the Newton method with a second-order approximation of $L(F^{t-1}+h)$ is used. The gradient step *h^t^* is selected so that *h^t^*(*x*) approximates −*g^t^*(*x,y*), where *g^t^*(*x,y*) = $\frac{\partial L(y,s)}{\partial s}$ with *s* = *F^t^*^−1^(*x*).

With the use of a least-squares approximation, the based predictor can be obtained as follows:

(10) $$h^{t}=\arg\min E{\left(-g^{t}(x,y)-h(x)\right)}^{2}$$

#### 2.4.2. Convolutional Neural Network Regressor

To predict urban LST, this study employs a convolutional neural network (CNN) that processes one-dimensional input features. A one-dimensional CNN is a specialized type of neural network for processing sequential data (e.g., time series and signals) [74]. This type of CNN employs convolutional layers that consist of tunable kernels across the input sequence [75]. These kernels enable the network to efficiently capture local patterns in the dataset. Given a one-dimensional vector *x* and a kernel filter *k*, the operation in a convolutional layer can be mathematically expressed as follows [74]:

(11) $$(x*k)(i)=\sum_{j=0}^{v-1}x(i+j)\cdot k(j)$$

where *x* and *k* represent the input data and the kernel, respectively; (x∗k)(i) denotes the convolution of the *x* and *k* at an index *i*; *v* is the size of the filter; and *k*(*j*) is the parameter of the kernel at index *j*.

The forward propagation of the model yields the output Z given an input *X*; this process can be described as follows [74]:

(12) $$Z=f_{A}(X*W+b)$$

where *W* and *b* are the tunable parameters (or weights) of the filter and the bias parameter, respectively.

A CNN model typically includes multiple hidden layers. This hierarchical structure serves as an automated feature engineering operator and allows a CNN to identify complex patterns governing the relationship between urban LST and its explanatory variables. For deep CNNs, a Rectified Linear Unit is often employed as the activation to model complex relationships in the data. Moreover, max pooling is used to reduce the dimensionality of the feature maps and retain the most significant features. Recent research has shown good capability of CNN in various complex function approximation tasks [76] and geospatial data analysis [77,78].

### 2.5. Model Evaluation

Accurate spatial modeling of LST is crucial because it guarantees the reliability of the machine learning models in generalizing the spatial variability of LST across the study area. These models can capture the complex interactions between explanatory variables, which influence the LST patterns. Reliable LST predictions directly support urban planning and the development of effective heat mitigation strategies by enabling the identification of hotspots, optimization of LULC configurations, and formulation of sustainable urban development plans.

Accordingly, this study employs four metrics to assess the performance of machine learning models in predicting urban LST: Root Mean Square Error (RMSE), Mean Absolute Percentage Error (MAPE), Mean Absolute Error (MAE), and the coefficient of determination (R^2^). These metrics help quantify the prediction accuracy of the models. The equations of these metrics are described in Table 3. In this table, *t_i_* and *y_i_* are the actual and predicted LST values of the *i*th data point, respectively. *N* represents the total number of data instances.

RMSE measures the average magnitude of prediction errors. The lower the values of RMSE, the more desired the prediction performance. Low MAPE indicates good accuracy, while higher values reflect larger average percentage errors. In practice, a MAPE below 10% often indicates a capable prediction model [79]. A value of MAE of 0 indicates perfect prediction performance. In addition, MAE is less sensitive to outliers than RMSE due to the fact that this metric relies on absolute errors instead of squared errors. The coefficient of determination (R^2^) ranges from 0 to 1. An R^2^ value of 1 implies that the model can explain all the variability in the dependent variable. Meanwhile, a low value of R^2^ indicates that the model poorly explains the variability in urban LST.

### 2.6. Shapley Additive Explanations (SHAP) for Feature Analysis

SHAP (Shapley Additive Explanations) [80,81] is a widely used method for interpreting the predictions of complex machine learning models by quantifying the contribution of each input feature to a specific prediction. This method is inspired by cooperative game theory, specifically the concept of Shapley values. These values provide a means to distribute the prediction outcomes among all features based on their individual contributions. Hence, SHAP can help attain local explanations (for individual predictions) as well as global explanations into feature importance across the entire dataset via aggregating individual estimations.

Identification of the influential factors is crucial for understanding the spatial distribution of LST. For effective urban planning and mitigation of urban heat stress, it is also important to acquire insights into how each variable affects LST. The SHAP method is highly appropriate for the task of interest since it can explicitly interpret model predictions by assigning an importance value to each explanatory variable in the GIS dataset.

Particularly, for tree-based models such as CatBoost, the TreeSHAP algorithm [82] is specifically developed to efficiently compute the SHAP values by harnessing the structure of decision trees. When predicting urban LST with machine learning models, SHAP highlights the features that strongly influence the predicted values. Moreover, the results of SHAP analysis can be conveniently summarized with an impact plot. This plot can help visualize the overall importance of each feature as well as the direction of its impact. Due to such advantages, SHAP has been increasingly utilized in urban heat stress studies [83,84,85,86].

### 2.7. Machine Learning-Based Causal Inference

In general, causal inference provides a framework to discover and quantify causal relationships in complex systems [40,87]. The inference process involves the following main steps:

(i) The causal problem is modeled by defining a graph that includes treatment variables (e.g., built-up density), outcome variables (urban LST), confounding factors, and mediators.

(ii) The causal effect is identified using criteria like backdoor adjustment to ensure that the effect can be estimated from observed data.

(iii) To guarantee the robustness of the analysis outcomes, refutation processes (e.g., placebo treatment tests) are conducted.

It is noted that the causal relationship can be expressed by directed acyclic graphs (DAGs) [88]. DAGs visually summarize causal assumptions by representing variables as nodes and causal relationships as directed arrows. This presentation is highly useful for the urban LST modeling task in which complex interactions exist among explanatory variables. Notably, machine learning can be applied in causal inference to model complex, non-linear relationships among treatment, outcome, and confounders [89,90,91]. This advanced data-driven approach can effectively deal with high-dimensional data and is able to capture sophisticated interactions among explanatory variables without pre-specified assumptions.

In this study, machine learning is utilized to model causal relationships between the governing factors and urban LST. The constructed model is then used to estimate the average treatment effect (ATE) for a variable of interest (e.g., green space density or built-up density). The libraries of DoWhy (https://github.com/py-why/dowhy) (accessed on 3 June 2025) and econml (Economic Machine Learning) (https://github.com/py-why/EconML) (accessed on 3 June 2025) are used to perform the machine learning-based causal inference processes.

## 3. Prediction Results

This study has prepared the GIS dataset used for geospatial modeling of urban LST. This dataset consists of 12 explanatory variables and 45,000 data points. They are organized into five main categories: topographic features, LULC, urban morphology, proximity features, and geographical compactness. For the case of CatBoost, the dataset was randomly divided into two subsets: a training set comprising 70% of the data and a testing set comprising the remaining 30%. The training set was used to fit the machine learning models and optimize model parameters, while the testing set was reserved for evaluating the models’ generalization capability on unseen data.

In order to normalize the explanatory variables, this study utilizes the Z-score method, which is expressed as follows:

(13) $$X_{Z}=\frac{X_{O}-\mu_{X}}{\sigma_{X}}$$

where *X_Z_* and *X_O_* denote the normalized and the original features, respectively, and *µ_X_* and *σ_X_* are the mean and standard deviation of the original feature, respectively.

To ensure the model’s reliability and prevent overfitting, the cross-validation approach [92] was employed during training. Specifically, five-fold cross-validation was performed within the training data. In this procedure, the training set was split into five equal-sized folds. During each of the five iterations, four folds were used to train the model, and the remaining fold served as a temporary validation set for model assessment. This process was repeated so that every fold was used once for validation. The performance metric of RMSE were averaged across all five folds to identify optimal hyper-parameters and ensure reliability of the model selection.

For the construction and evaluation of the CNN model, the original dataset is randomly partitioned into three subsets: a training set (60%), a validation set (10%), and a testing set (30%). The training set is used to fit the model. Throughout training, model performance is monitored on the separate validation set. The validation error, calculated after each epoch using the validation samples, serves as an indicator for detecting overfitting and for guiding model tuning. To further prevent overfitting, the training procedure utilizes early stopping: the learning process is halted automatically if the validation error ceases to improve. This framework ensures that the model parameters are set at the point of optimal generalization capability. The CNN’s hyper-parameters—including the number of convolutional layers and the maximum number of training epochs—are determined through multiple trial-and-error runs, where their suitability is assessed by the validation set performance.

The hyper-parameters of CatBoost are selected as follows: number of boosting iterations = 600, the maximum tree depth = 8, the learning rate = 0.15, and the *L*_2_ leaf regularization coefficient = 0.0001. The CNN model consists of three hidden layers; each layer contains 128 convolutional filters. The maximum number of training epochs is 100. The learning rate of CNN is set to be 0.001. The adaptive moment estimation (Adam) optimizer [93] is used to train the CNN model with a batch size of 256.

**Figure 11:**
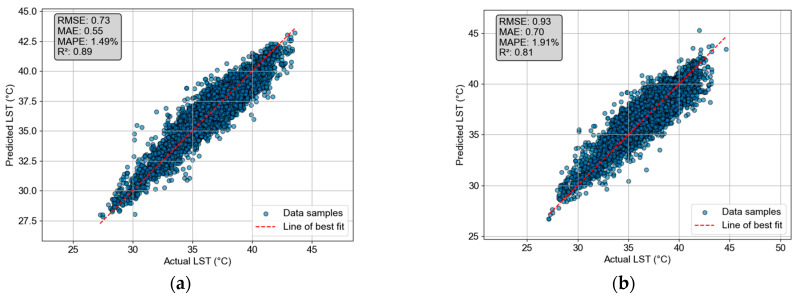
Prediction results: (**a**) CatBoost and (**b**) CNN.

The prediction performance of the CatBoost and CNN models was summarized in Table 4. As mentioned earlier, the metrics of RMSE, MAPE, MAE, and R^2^ are employed to evaluate the models’ performance. In the training phase, CatBoost consistently demonstrated superior performance compared to the CNN; the gradient boosting machine achieved a lower RMSE (0.50 vs. 0.68), MAPE (1.03% vs. 1.40%), and MAE (0.38 vs. 0.51), as well as a higher R^2^ value (0.95 vs. 0.90). In the testing phase, a slight decline in performance was observed with the two models. Nevertheless, CatBoost still delivers satisfactory performance with an RMSE of 0.73 compared to the value of 0.93 yielded by CNN. CatBoost also achieved a lower MAPE (1.49% vs. 1.91%) and MAE (0.55 vs. 0.70). This model attains a good R^2^ value of 0.89; this fact implies that CatBoost can explain 89% of the variation in the urban LST recorded in the urban center. The detailed prediction results of the two machine learning models are visualized via the scatter plots in Figure 11. As shown in the figure, the prediction outcomes attained by CatBoost are closer to the line of best fit compared to those yielded by CNN. Hence, it can be concluded that CatBoost is highly suitable for the task of interest.

**Figure 12:**
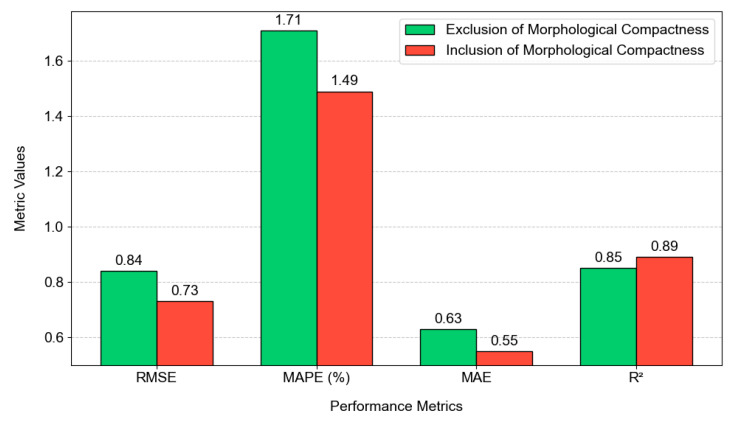
Impact of urban geographical compactness on urban LST prediction.

Furthermore, to demonstrate the critical role of geographical compactness in urban LST modeling, this study compares the performance of CatBoost models with and without the use of the geographical compactness features (i.e., built-up cluster compactness and green space cluster compactness). To support hypothesis testing, the processes of training and prediction with the collected GIS dataset are repeated 20 times. In each run, 30% of the whole dataset is used as the testing samples; the rest of the dataset is used to train the CatBoost models. Figure 12 summarizes the comparative analysis of CatBoost performance. In general, the analysis reveals a substantial improvement when geographical compactness features are incorporated into urban LST prediction. CatBoost without geographical compactness achieved an R^2^ value of 0.85, RMSE of 0.84, MAE of 0.63, and MAPE of 1.72%. Meanwhile, the CatBoost model using the compactness features demonstrated superior performance in all metrics, with an R^2^ of 0.88, RMSE of 0.74, MAE of 0.56, and MAPE of 1.52%.

**Figure 13:**
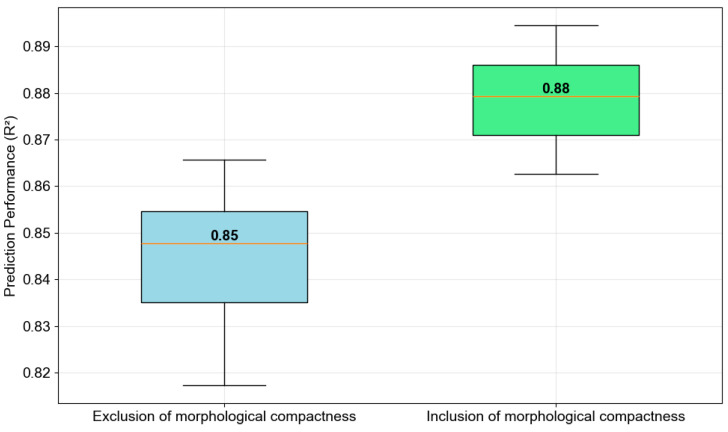
The modeling performance in terms of R^2^.

Figure 13 further describes the performance of the CatBoost models using two feature sets. The box plots provided in this figure help illustrate the performance enhancement gained by the geographical compactness. Apparently, the inclusion of geographical compactness features not only increases the median R^2^ value from approximately 0.85 to 0.88 but also reduces the variability in model predictions, as shown by the tighter interquartile range. Moreover, to demonstrate the statistical significance of this performance difference, this study relies on the Wilcoxon Signed-Rank Test (WSRT). The WSRT is a non-parametric hypothesis test that is widely used for prediction result comparison. The WSRT yielded a test statistic of 0.00 and a *p*-value of 0.0000019. With a *p*-value < 0.05, the null hypothesis of equal performance can be rejected, and it is reliable to confirm that the two models perform differently. The test outcome indicates that geographical compactness features (built-up compactness and green space compactness) provide valuable spatial information that enhances the CatBoost model’s ability to capture the functional mapping between urban LST in the urban center and its influencing factors.

**Figure 14:**
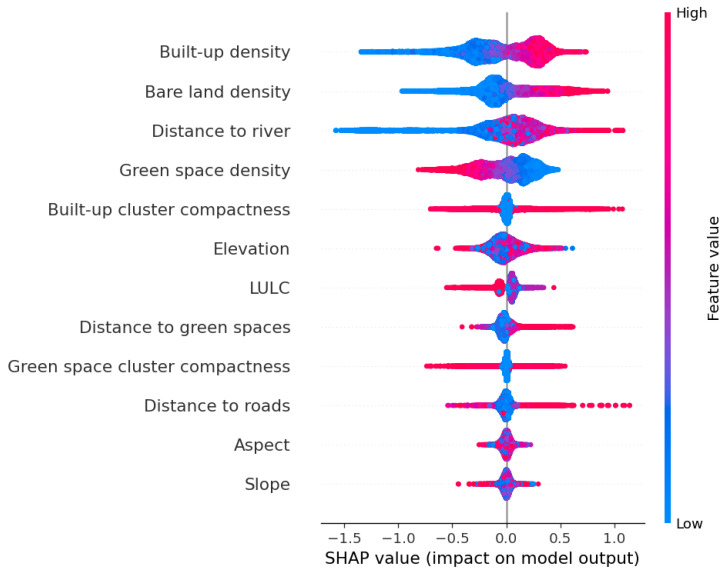
Significance of independent variables based on the SHAP index.

This study relies on SHAP to investigate the importance of features used in the CatBoost model. As shown in Figure 14, SHAP reveals that built-up density is the most influential factor, followed by bare land density, distance to river, green space density, and built-up cluster compactness. These features exhibit the largest spread of SHAP values and imply strong effects on the model’s predictions. Elevation, LULC, distance to green spaces, and green space cluster compactness also demonstrate notable contribution. However, their impacts are less than those of the aforementioned variables.

Distance to roads shows moderate importance. This finding suggests that proximity to roads has a certain influence on the spatial variation of LST in the study area, but this feature does not impose a dominant effect. This result can be explained by the fact that the impact of roads may be partly mitigated or overshadowed by other influential factors such as built-up density, LULC composition, and the compactness of urban clusters. Furthermore, the spatial configuration of roads can be interspersed with green spaces or sparsely built-up land. This fact further reduces the overall influence of roads on LST patterns.

Meanwhile, aspect and slope have relatively minor impacts on LST in the region. The small SHAP values obtained for aspect and slope suggest that variations in these topographic features exert limited influence on LST compared to other factors. This result may reflect the fact that aspect and slope conditions in the study area do not vary substantially to yield strong microclimatic differences. Accordingly, their effects are dominated by those of the urban morphological features and LULC patterns.

**Figure 15:**
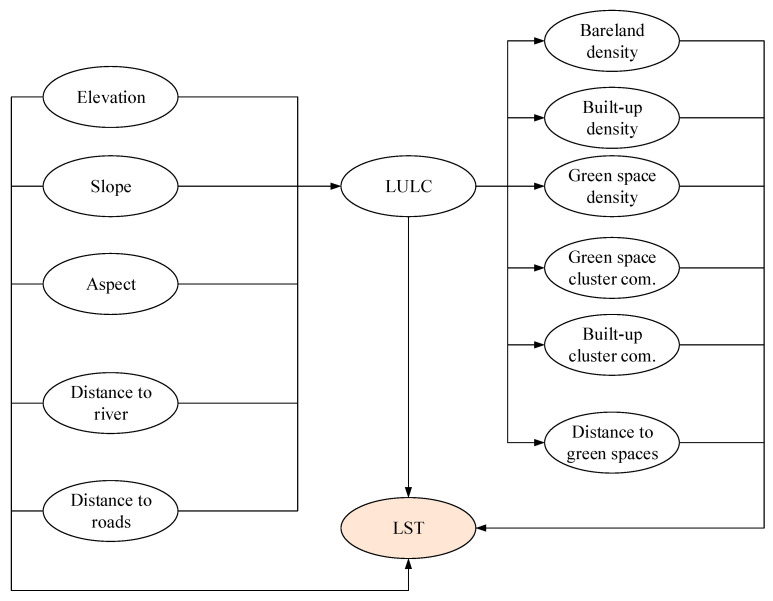
Causal inference graph.

Since CatBoost has been confirmed to be a reliable method for spatial prediction of urban LST, this study employs this machine learning method in a causal inference framework to estimate average treatment effects of urban features on LST. The model utilizes the estimator that implements double machine learning for confounding control and causal effect computation. The causal relationships for this study are encoded in a DAG shown in Figure 15. Using this DAG, the outcome variable is urban LST. The green space density, bare land density, built-up density, distance to river, distance to roads, and distance to green space are selected as treatment variables to compute their effects on the outcome. Moreover, the reliability of the causal inference results is confirmed via placebo tests. The inference outcomes are summarized in Table 5.

As shown in the table, the integrated framework with CatBoost estimated significant causal effects of the urban features of interest on LST. It is noted that the density features are estimated for a 90 × 90 m grid cell. The result indicates that bare land density increased LST by 0.0404 °C per 1% increment, and built-up density raised the value of the outcome variable by 0.0623 °C per 1% increase. The results also show that increasing green space density reduces LST, with a 1% increase in green space density associated with a decrease of 0.0087 °C. The proximity variables also reveal that distances to river and roads only result in minor changes in LST. Moreover, the placebo tests also indicate the validity of the causal inference with *p*-value > 0.05. These tests help confirm the true causal effect of the treatment on the outcome.

## 4. Discussion

### 4.1. Machine Learning Performance

Via the experimental result, CatBoost has demonstrated its capability of delivering accurate prediction of urban LST. Notably, the gradient boosting machine can explain up to 89% of the variation in the target output. Its performance has significantly surpassed that of the CNN regressor. This fact can be explained by the excellent ability of CatBoost in handling large-scale and high-dimensional data with complex non-linear relationships [94]. These characteristics are typical in GIS datasets. CatBoost supports the learning process for both real-value and categorical variables and is capable of capturing the complex interactions among urban features.

Another notable advantage of CatBoost is its handling of overfitting; this feature is achieved through techniques of ordered boosting and regularization. The model gains high prediction accuracy for both training (R^2^ of 0.95) and testing samples (R^2^ of 0.89). These results indicate that CatBoost not only provides a good fit to the training data but also reliably predicts LST for unseen input features. Additionally, when being used with SHAP, CatBoost is capable of delivering both high accuracy and interpretability [95]. This feature is particularly useful in spatial modeling of urban LST.

Notably, Landsat surface temperature retrievals are subject to known errors influenced by atmospheric conditions and the presence of cloud shadows [96]. The validation results reported in [97] show a mean error of −0.267 °C and a standard deviation of 0.900 °C from an analysis of 259 cloud-free scenes. Accordingly, it should be noted that the modeling errors observed in machine learning approaches not only reflect the predictive capacity of the models themselves but also are constrained by the accuracy limits of the underlying LST data. Thus, even if a machine learning model is well trained and highly optimized, its minimum achievable error cannot be lower than the fundamental uncertainty of the satellite-derived LST product.

Based on the model’s performance, it can also be seen that the inclusion of geographical compactness is critical for accurate urban LST modeling. This result has been confirmed by the WSRT. Notably, the geographical compactness captures the spatial configuration and aggregation patterns of urban landscape [33]. Therefore, geographical compactness can be helpful for modeling the thermal behavior in the study area. The result in this study generally aligns with the findings in previous works [98,99,100], which pointed out the relationship of urban compactness and thermal behaviors.

**Figure 16:**
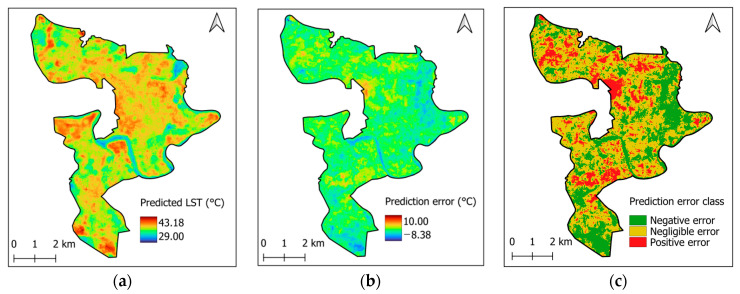
Prediction result of CatBoost: (**a**) LST mapping, (**b**) prediction error, (**c**) spatial pattern of prediction error classes.

Figure 16a demonstrates the LST mapping yielded by the CatBoost model. The spatial distribution of prediction errors and their categories are shown in Figure 16b and Figure 16c, respectively. Based on the model error in this study and the validation results reported in [97], the range of 0 ± 1.0 °C is defined as representing negligible errors. Notably, a positive prediction error indicates that the model underestimates the actual LST value, while a negative prediction error implies an overestimation. It was found that in 14.63% of the cases, the model underestimated the actual LST values. Moreover, the analysis based on Moran’s I test yielded a Moran’s I value of 0.66 and a *p*-value = 0.001 < 0.05. This outcome indicates a strong positive spatial autocorrelation among the sites where underestimations occur. In other words, urban areas with underestimated LST are not randomly dispersed but tend to cluster in specific regions, as illustrated in Figure 16c.

Underestimation of LST can have undesirable consequences in the study area. Most importantly, it may lead to a failure to recognize local heat stress, which is particularly critical for urban planning and human health risk assessments. If areas with high LST are not correctly identified, heat mitigation strategies may not be effectively implemented. Furthermore, underestimating LST could hinder the accurate evaluation of the UHI effect. Therefore, caution is needed when applying the model’s prediction results in areas where LST is underestimated, as this may affect the reliability of subsequent analyses and decision-making.

### 4.2. Implications for Urban Heat Stress Mitigation

The findings of this study can be used to suggest several recommendations for mitigating urban heat stress in the study area. SHAP-based interpretation and CatBoost prediction reveal that built-up density is the most influential factor in reducing LST. High built-up density has been proven to be a major contributor to increased urban LST and intensified heat stress in cities [14,101,102]. This result accentuates the need to avoid excessive urban densification without adequate urban green spaces. Density regulation is also recommended in hot spots associated with the high UHIEI class. The implementation of green roofs and optimized urban layouts should be encouraged to enhance thermal comfort.

Since green spaces have demonstrated strong cooling effects via SHAP and causal inference analyses, increasing both the quantity and quality of urban greenness should be a top priority for urban planners in the study area. This evidence is further supported by the finding that compact green space clusters, as opposed to fragmented or sprawling patches, maximize cooling benefits. This finding is in agreement with previous studies that investigated the spatial configuration and compactness of green areas [103,104].

SHAP analysis also highlights bare land density as a critical contributor to the rise in LST. Accordingly, prioritizing the conversion of bare land areas into green spaces is strongly recommended. Moreover, the cooling effect of rivers suggests that urban planners in the study area should prioritize maintaining and enhancing public access to these features. This is because their cooling effects can extend beyond their boundaries. However, the relationship between built-up compactness and urban LST can be complex and context-dependent. In certain cases, sprawling urban forms or small urban patches can actually intensify heat stress, as highlighted in [100].

### 4.3. Limitations and Future Works

Although this study has developed and verified a novel data-driven framework for spatial modeling of LST, several limitations should be acknowledged. First, the characterization of urban morphology relied primarily on two-dimensional metrics of built-up density, bare land density, and green space density. The influence of three-dimensional (3D) urban form, such as building height, was not assessed due to the unavailability of high-resolution and up-to-date 3D spatial data in the region. Recent research confirmed that 3D compactness could considerably affect the cooling effect of green space and the UHI phenomenon [34,105,106]. Therefore, the absence of these 3D spatial features may unavoidably limit the model’s capability in accurately representing the relationship between urban morphology and LST.

In this study, green spaces were identified based on general vegetation detection using Sentinel-2 spectral bands. Different types of vegetation cover were not distinguished. Additionally, this study has not considered other important metrics for landscape configuration, such as diversity and connectivity of green spaces. These factors have been shown to influence LST by affecting the extent and efficiency of the cooling capability of urban greenness [99,106]. Moreover, socioeconomic factors—including demographic variables and the government’s policies in land use planning—were not incorporated into the current framework. The exclusion of these variables may limit the ability of the proposed method to fully capture the drivers of heat stress variations in the region.

Additionally, the use of Landsat 8 imagery with a spatial resolution of 30 m, while suitable for regional-scale analyses, inevitably introduces some degree of spatial generalization. At this resolution, mixed pixels are likely to occur. This fact can reduce accuracy in capturing local variations in LST. According to the spatial pattern of prediction error sign, there is a strong spatial autocorrelation among the areas associated with underestimated LST. Hence, there may be underlying factors, possibly related to local urban morphology, LULC, microclimate, or other unexplored site-specific variables, that lead the model to underestimate LST in these areas. Notably, the influence of wind and air circulation—which are well recognized for their impact on the intensity and distribution of UHI—was not considered in the current work. Furthermore, for constructing the variable of distance to road, all roads were treated as a single category, without distinguishing between surface materials or road classifications. As a result, potential variations in the impact of different types of roads and traffic volumes on LST may not be fully captured.

To address the aforementioned limitations and further improve the predictive capability of urban LST modeling, several future research directions are recommended:

(i) Future works should incorporate more diverse urban morphology metrics. The inclusion of 3D data can help to attain a more accurate representation of the urban thermal behavior.

(ii) More sophisticated metrics related to urban green spaces should be employed to better capture the effects of spatial configuration and quality of vegetation on cooling efficiency. Additionally, detailed field surveys and higher-resolution remote sensing data should be employed to accurately categorize various forms of vegetation. This would allow for a more precise evaluation of urban green spaces and their individual contributions to the spatial variation of LST.

(iii) Socioeconomic factors and government policies should be included to enhance the holistic understanding of the drivers of urban heat stress in the study area.

(iv) The exploration of other advanced machine learning architectures can be helpful to further improve the model’s predictive accuracy and generalizability.

(v) Future research should also investigate and benchmark additional state-of-the-art clustering algorithms appropriate for geospatial data. Exploring advanced methods—such as density-based spatial clustering, hierarchical clustering, and deep learning-based techniques—could uncover more sophisticated spatial patterns and urban structures. Incorporating these approaches may enhance the identification of meaningful clusters, improve the representation of urban form, and enable more accurate modeling of urban LST variation.

(vi) High-resolution remote sensing data or ground-based observations can be utilized to improve the accuracy of LST predictions.

(vii) Further analyses of the clustered regions of underestimated LST are critical for deeper understanding of the model limitations. Incorporating additional spatial predictors and refining the machine learning model’s parameters can enhance the model’s ability to accurately capture the spatial heterogeneity of urban LST in the study area. These improvements may help reduce spatially biased prediction errors.

(viii) Additional fieldwork should be carried out to obtain accurate descriptions and assessments of local wind characteristics and air exchange patterns. These data can help achieve a deeper understanding of the UHI effect in the study area.

(ix) Another promising future direction for this work is to incorporate potential solar radiation (PSR) into geospatial modeling of urban LST. By taking into account PSR—which integrates slope, aspect, latitude, and solar geometry—the predictive capability of machine learning models for LST estimation can be enhanced.

(x) Data on different road types and traffic volumes should be integrated into the GIS dataset to enable a more accurate assessment of the effects of roads on urban LST.

(xi) To further improve the interpretability and effectiveness of the current framework, additional analyses of model errors and their underlying causes should be conducted. In particular, it would be beneficial to examine the model’s performance across different combinations of the explanatory variables.

## 5. Concluding Remarks

This study has introduced a data-driven framework for the spatial analysis and prediction of urban LST, a rapidly urbanizing area in Vietnam’s Central Highlands. The framework is a novel combination of remote sensing data, geospatial analyses, and advanced machine learning methods, specifically CatBoost and CNN. The current work demonstrates the effectiveness of machine learning and big data analysis in unraveling the complex relationships between urban form and thermal behavior. The CatBoost model exhibited an outstanding predictive performance. This model can explain 89% of the variance in LST. This outcome accentuates the strength of this gradient boosting algorithm for spatial modeling of urban heat stress, especially when dealing with complex and non-linear relationships among explanatory variables.

The incorporation of urban compactness metrics, combining *k*-means clustering and the Polsby–Popper Compactness index, has been shown to significantly enhance model accuracy. Moreover, the use of SHAP provided valuable details about feature importance. It revealed the significance of built-up density, bare land density, distance to river, green space density, and built-up cluster compactness. This study also relied on a causal inference analysis to further clarify the effects of urban morphological and proximity features on LST. Accordingly, the proposed framework presents a practical and interpretable tool for supporting sustainable urban development in the southern region of Quang Ngai Province, Vietnam, as well as in adjacent urban areas in Vietnam’s Central Highlands. By providing data-driven insights into the key factors of urban heat stress, this research can help enhance the sustainable planning process, especially in the context of rapid urbanization and climate change.

## Figures and Tables

**Table 1:** The employed remote sensing data.

Dataset	Filtered Date	Bands	Resolution	Number of Images in Collection
Landsat 8	1 January 2024–31 March 2024 1 December 2024–31 December 2024	SR_4, SR_5, and ST_B10	30 m	7
Sentinel-2	1 January 2024–31 December 2024	B2, B3, B4, B5, B6, B7, B8, B8A, B11, B12	10 m (B2, B3, B4, and B8) 20 m (B5, B6, B7, B8A, B11, and B12)	29
NASA SRTM Digital Elevation 30 m		Elevation	30 m	1

**Table 2:** Explanatory variables in the GIS dataset.

Category	Variables	Data Source
Topographic features	Elevation	NASA SRTM Digital Elevation 30 m
	Slope	
	Aspect	
LULC	LULC	RF-based classification using Sentinel-2’s spectral bands
Urban morphology	Bare land density	Computed from LULC maps using morphological mean filters
	Built-up density	
	Green space density	
Proximity features	Distance to green spaces	Calculated using geometry objects and distance measurements
	Distance to river	
	Distance to roads	
Geographical Compactness	Built-up cluster compactness	Computed using *k*-means clustering and the Polsby–Popper index
	Green space cluster compactness	

**Table 3:** Performance measurement metrics.

Indices	Notation	Equations	Explanation
Root Mean Square Error	RMSE	$RMSE=\sqrt{\frac{1}{N}\sum_{i=1}^{N}{\left(y_{i}-t_{i}\right)}^{2}}$	This index computes the standard deviation of prediction errors and indicates the average differences between actual and predicted LST values.
Mean Absolute Percentage Error	MAPE	$MAPE=\frac{100}{N}\times \sum_{i=1}^{N}\frac{\left|y_{i}-t_{i}\right|}{y_{i}}$	MAPE expresses the prediction errors as a percentage of actual values; this index shows the relative magnitude of the error.
Mean Absolute Error	MAE	$MAE=\frac{1}{N}\times \sum_{i=1}^{N}\left|y_{i}-t_{i}\right|$	This index computes the average absolute value of the errors. Compared to RMSE, MAE is more robust to outliers in the dataset.
Coefficient of determination	R^2^	$R^{2}=1-\frac{\sum_{i=1}^{N}{\left(t_{i}-y_{i}\right)}^{2}}{\sum_{i=1}^{N}{\left(t_{i}-\bar{t}\right)}^{2}}$	This metric represents the proportion of variation in urban LST explained by the model.

**Table 4:** Prediction performance.

Phase	Metrics	CatBoost	CNN
Training	RMSE	0.50	0.68
	MAPE (%)	1.03	1.40
	MAE	0.38	0.51
	R^2^	0.95	0.90
Testing	RMSE	0.73	0.93
	MAPE (%)	1.49	1.91
	MAE	0.55	0.70
	R^2^	0.89	0.81

**Table 5:** Estimated Causal Effects.

Variable	Unit	ATE	Placebo Test *p*-Value
Bare land density	%	0.0404 °C	0.1336
Built-up density	%	0.0623 °C	0.4002
Greenspace density	%	−0.0087 °C	0.4534
Distance to green spaces	m	0.0043 °C	0.3611
Distance to river	m	0.0001 °C	0.4057
Distance to roads	m	0.0002 °C	0.4389

## Data Availability

The data that support the findings of this study are available from the corresponding author upon reasonable request.
